# Diagnostic accuracy of lung ultrasonography combined with procalcitonin for the diagnosis of pneumonia: a pilot study

**DOI:** 10.1186/s13089-016-0054-8

**Published:** 2016-11-09

**Authors:** Peiman Nazerian, Gabriele Cerini, Simone Vanni, Chiara Gigli, Maurizio Zanobetti, Maurizio Bartolucci, Stefano Grifoni, Giovanni Volpicelli

**Affiliations:** 1 Department of Emergency Medicine, Careggi University Hospital, Largo Brambilla 3, 50134 Florence, Italy; 2 Radiology Department, Careggi University Hospital, Florence, Italy; 3 Department of Emergency Medicine, San Luigi Gonzaga University Hospital, Turin, Italy

**Keywords:** Biomarkers, Diagnosis, Pneumonia, Ultrasound, Procalcitonin

## Abstract

**Background:**

The diagnostic value of lung ultrasonography (LUS) and procalcitonin (PCT) in the diagnosis of lung infections is known. No studies evaluated the combination of LUS and PCT for the diagnosis of pneumonia in the emergency department (ED). We evaluated the diagnostic accuracy of the combination of LUS and PCT in the diagnosis of pneumonia.

**Methods:**

Patients with respiratory symptoms of unexplained origin who underwent a chest CT in ED were included in the study if PCT assay was available. LUS was performed before CT and was targeted to the detection of lung consolidations with the morphologic features of pneumonia. A PCT assay was performed at presentation, and cut-off of 0.25 and of 0.5 ng/ml were used to rule-out and rule-in pneumonia. The final diagnosis of pneumonia was established by independent clinicians, on the basis of clinical chart review including CT results.

**Results:**

We enrolled 128 patients and pneumonia was the final diagnosis in 61 (47.7%). In 38 patients (29.7%) LUS and PCT were negative (PCT < 0.25 ng/ml). The overall accuracy, sensitivity and negative predictive value of LUS/PCT were 88.8, 96.7 and 94.7% respectively. Sensitivity of the LUS/PCT test was significantly superior to LUS alone (85.2%) and PCT alone (73.8%) (*p* < 0.05 for both). Specificity and positive predictive value of the combination of positivity of LUS/PCT (PCT > 0.5 ng/ml) were 94% and 83.3% respectively. Specificity of LUS/PCT was not significantly different to LUS alone (88.1%) (*p* = 0.125).

**Conclusions:**

The sensitivity of the combination of LUS with PCT for the diagnosis of pneumonia was significantly superior when compared with the sensitivity of LUS and PCT alone.

## Background

Pneumonia is an infection that involves alveoli, distal airways, and interstitium leading to lung inflammatory consolidations and is one of the major infection-related cause of death in developed countries [1, 2].

The mainstay of the diagnosis is based on symptoms and signs consistent with acute infection of the lower respiratory tract associated with the visualization of at least one pulmonary consolidation at chest imaging. For the purpose of imaging the consolidation, chest computed tomography (CT) is the diagnostic gold standard. Unfortunately CT can’t be used routinely because of high cost, radiation exposure and scarce availability in low resources settings [3]. Thus, in clinical practice, chest radiography (CXR) represents the standard of care and is mostly used to diagnose pneumonia. However, in patients evaluated in the emergency department (ED), CXR showed a poor sensitivity (43.5%) when compared to CT [4]. Therefore, reliance on CXR to diagnose pneumonia may lead to significant rates of misdiagnosis.

Lung ultrasonography (LUS) is an emerging bedside diagnostic tool with a sensitivity of 94% and a specificity of 96% for the diagnosis of pneumonia according to a recent metanalysis [5]. Procalcitonin (PCT) expression in parenchymal tissue is induced by bacterial infection and its concentrations have been successfully used to diagnose and guide antibiotic therapy in lower respiratory tract infections [6, 7].

No previous studies have evaluated the accuracy of a combination of LUS and PCT for the diagnosis of pneumonia in the ED. Thus, the aim of our study was to assess the diagnostic accuracy of the combination of LUS with PCT when the final diagnosis was supported by a chest CT imaging.

## Methods

### Study subjects and design

This is a prospective accuracy study, in which patients enrolled in a registry from December 2011 to August 2012 were included if PCT level at admission was available [8]. Patients were recruited in the ED with an annual census of 130,000 visits. The study was approved by the local institutional review board. Written informed consent was obtained for inclusion in the study.

Consecutive patients aged >18 years with at least one unexplained respiratory complaint among dyspnea, chest pain, cough or hemoptysis with or without fever, for which the attending emergency physician ordered a chest CT were considered in the study. PCT was ordered by the attending emergency physician, independent from the study protocol, when a infective cause was suspected.

### Methods

Lung ultrasonography was performed before and within 3 h from chest CT by one of eight sonographer investigators who participated to the study. The investigators were four internal and emergency medicine staff physicians with at least 5 years experience on point-of-care emergency ultrasonography and four resident physicians (two in Internal Medicine and two in Emergency Medicine) with at least 6 months training in emergency ultrasound. The investigators were aware of the presenting symptoms and the evident physical signs, but were blinded to all the other general clinical information including any radiologic finding and laboratory results. The following multi-probe machines were used: MyLab30 Gold (Esaote, Genova, Italy) and HD7 (Philips, Amsterdam, Holland). LUS was performed by using a 4–8 MHz linear probe or a 3.5–5 MHz curved array probe. The lung was examined by longitudinal and oblique scans on anterior, lateral and posterior chest. The patient was preferably examined in the sitting position. When this latter position could not be maintained, due to critical clinical conditions or poor compliance, the examination was performed in the supine or semi-recumbent position. The posterior areas were scanned in the sitting position or, when not feasible, by turning the patient in the lateral decubitus on both sides. The LUS examination was targeted to the detection of typical subpleural lung consolidations with tissue-like or anechoic pattern and blurred, irregular margins with associated focal B-lines [9]. The consolidations due to infection usually show dynamic air bronchograms (branching echogenic structures with centrifuge movement with breathing) or multiple hyperechogenic lentil-sized spots, due to air trapped in the small airways. LUS was considered positive when at least one consolidation showing the above-described features was detected. Pleural effusion could be associated with consolidation and has been interpreted as an auxiliary sign, but its detection was not considered individually as sign of suspected pneumonia.

Chest radiography was performed using a Practix 300 Bucky diagnostic equipment (Philips Medical Systems, Hamburg, Germany) by posterior-anterior and lateral views in the upright patients and anterior-posterior view in the supine patients, following standardized hospital diagnostic protocol. The film was digitally reviewed by an expert radiologist blinded to the results of LUS and CT. The radiologist had the possibility to review also previous CXR, when available, as part of standard clinical care. The radiologist was asked to detect and locate the opacities that might correlate with the diagnosis of pneumonia. CXR was considered positive when at least one typical consolidation was visualized.

Procalcitonin, requested by the treating physician at ED presentation for clinical reason, was measured using an automated Enzyme-Linked Fluorescent Assay (ELFA): VIDAS^®^ B.R.A.H.M.S PCT™ (Vidas, BioMerieux). Samples were processed by personnel blinded from any patient data. We considered two PCT cut-off of 0.25 and 0.5 ng/ml based on previous studies [6, 7, 10–12]. These studies suggested that a PCT level <0.25 ng/ml can be used to rule out bacterial pneumonia in patients with suspected respiratory tract infections and a PCT level >0.5 ng/ml is considered to rule in bacterial pneumonia and to support antibiotic therapy. Patients that didn’t consent or didn’t undergo LUS within the time limit were excluded from the study. Patients in which PCT was not requested in ED were also excluded.

Chest CT was performed by one Somatom Definition AS128 (Siemens, Erlangen, Germany), only for clinical purposes independent from the study protocol. An expert radiologist, other than the attending radiologist and blinded to LUS and CXR results, reviewed the studies investigating one or more consolidations related to pneumonia, defined as homogeneous increase in pulmonary parenchymal attenuation obscuring the margins of vessels and airway walls, with or without air bronchograms [13].

The final diagnoses of pneumonia were determined by two expert internal medicine physicians, blinded to CXR, LUS and PCT results, who independently reviewed all available clinical data including CT data and medical records for hospitalized patients. In case of discordance, a third senior physician adjudicated the diagnosis.

### Statistical analysis

The sample size was calculated considering a prevalence of pneumonia of 50% among suspected patients in ED and a sensitivity of LUS for the diagnosis of pneumonia when chest CT was used as gold standard of 80% [8]. Wanting to prove that PCT increased the sensitivity of 10% and considering a type I error of 5% and a power (1-ß) of 80%, it was estimated that this would require approximately 120 patients.

Data points are expressed as mean ± standard deviation (SD). The unpaired Student’s *t* test was used to compare normally distributed data. Fisher’s exact test was used for the comparison of non-continuous variables expressed as proportions.* p* < 0.05 indicates statistical significance. All * p* values are two sided. The diagnostic performance of LUS, CXR and PCT and the combination of LUS and CXR with PCT was assessed by calculating sensitivity, specificity, positive predictive value, negative predictive value and likelihood ratios. The extended McNemar and the McNemar tests were used to evaluate if there was a significant difference in the accuracy, sensitivities and specificities of LUS, CXR, PCT and the combination of imaging tests with PCT [14]. Receiver operating characteristic (ROC) curves were constructed to assess the sensitivity and specificity of white blood cells count (WBC), PCT and multivariable models to compare the ability of LUS and CXR combined with PCT to predict pneumonia. Calculations were performed by using SPSS statistical package (version 17.0; IBM).

## Results

### Baseline characteristics

**Fig. 1:**
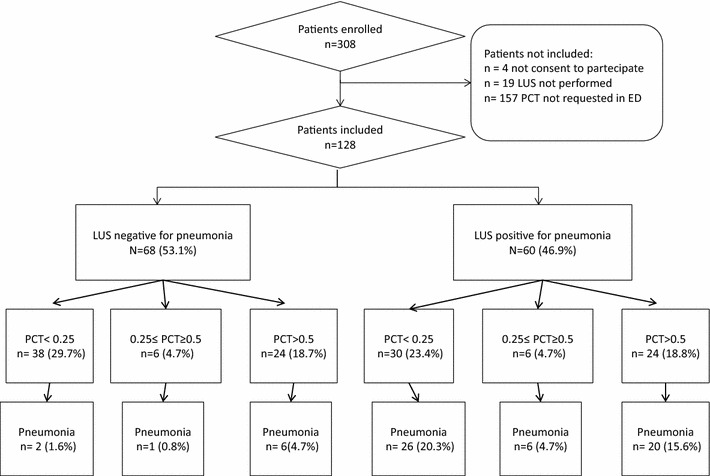
Characteristics of patients according to the presence or absence of pneumonia. *LUS* lung ultrasonography; *PCT* procalcitonin

A total of 308 patients with respiratory complains underwent chest CT in the ED during the study period. Four patients did not consent to participate. In 19 patients LUS was not performed before chest CT or within the time limit. In 157 patients PCT was not requested by the attending emergency physician at presentation in the ED. Thus, 128 patients were included in the final analysis (Fig. 1). These patients had a mean age of 70.7 ± 15.8 years (range 23–100) and 64 (50%) were female. Pneumonia was the final diagnosis in 61 patients (47.7%). The patients’ characteristics according to the presence or absence of pneumonia are shown in Table 1. Alternative diagnoses in patients without pneumonia are reported in Table 2.

**Table 1:** Characteristics of the study population according to final diagnosis

	Pneumonia (*n* = 61)	No pneumonia (*n* = 67)	*p* value
Mean age ± SD, y	69.8 ± 18.8	71.6 ± 12.6	0.51
Women	25 (41%)	39 (58.2)	0.076
*Medical history of lung disease*			
Chronic obstructive pulmonary disease	7 (11.5)	10 (14.9)	0.611
Lung cancer	2 (3.3)	5 (7.5)	0.444
Heart failure	11 (18)	14 (20.9)	0.824
Previous pulmonary embolism	1 (1.6)	3 (4.5)	0.621
*Signs and symptoms at presentation*			
Dyspnea	44 (72.1)	49 (73.1)	1
Cough	26 (42.6)	12 (31.6)	0.003
Pleuritic chest pain	8 (13.1)	8 (11.9)	1
Shock/hypotension	17 (27.9)	13 (19.4)	0.3
SBP ± SD, mmHg	121.7 ± 30.5	124.9 ± 37.3	0.6
Heart rate >100 bpm	42 (68.9)	30 (44.8)	0.008
Respiratory rate/min	26.1 ± 8.2	23.3 ± 6.8	0.2
PaO_2_/FiO_2_ ± SD	292.6 ± 83.9	309.5 ± 94	0.29
Temperature >37.8 °C	31 (50.8)	22 (32.8)	0.049
White blood cells count/mm^3^	12,494 ± 10,130.3	11,906 ± 6657	0.7
*Mechanical invasive ventilation*	4 (6.6)	6 (9)	0.747

Data are given as no. (%) or mean ± standard deviation (SD)

*SD* standard deviation; *SBP* systolic blood pressure; *PaO*_*2*_ arterial oxygen pressure express in mmHg; *FiO*_*2*_ fraction of inspired oxygen; *bpm* beats/min; *ED* emergency department

**Table 2:** Final diagnosis in the study patients

Diagnosis	No (%)
Pneumonia	61 (47.7)
Pulmonary embolism	21 (16.4)
Heart failure	9 (7)
Pleural effusion	8 (6.3)
Sepsis with no pulmonary involvement	8 (6.2)
Chronic obstructive pulmonary disease	4 (3.1)
Acute coronary syndrome	3 (2.3)
Tachyarrhythmia	1 (0.8)
Respiratory failure in pulmonary malignancy	1 (0.8)
Pericardial effusion/pericarditis	1 (0.8)
Miscellaneous	11 (8.6)

Data are given as no. (%) unless otherwise indicated

### Main results

Lung ultrasonography was feasible in all patients, but in 11 cases (8.2%), included in the study, pulmonary examination was limited only to the anterior-lateral areas mainly due to uncooperative patients. LUS was positive for at least one consolidation in 60 patients (46.9%). Fifty-two (86.7%) out of 60 patients with positive LUS had a final diagnosis of pneumonia (Fig. 1). LUS was false positive in eight cases (6.2%) and false negative in nine patients (7%). In three out of nine (33%) false negative cases pulmonary examination was limited only to the anterior-lateral areas. The sensitivity and the specificity of LUS were 85.2% (95% CI 73.8–93%) and 88.1% (95% CI 77.8–94.7%) respectively.

Procalcitonin median in patients with and without pneumonia were 0.3 and 0.1 ng/ml respectively (*p* = 0.05). The sensitivity and the specificity of PCT ≥ 0.25 ng/ml were 73.8% (95% CI 61–84.2%) and 47.8% (95% CI 35.4–60.3%) respectively, whereas the sensitivity and the specificity of* PCT* > 0.5 ng/ml were 42.6% (95% CI 30–55.9%) and 67.2% (95% CI 54.6–78.1%) respectively.

Chest radiography was performed with the patient in the up-right position in 29 (23%) cases. Thirty-seven out of 45 patients (82.2%) with positive CXR had a final diagnosis of pneumonia. CXR showed false positive examination in 8 (6.2%) patients and false negative examination in 24 (18.7%) patients. The sensitivity and the specificity of CXR were 60.7% (95% CI 47.3–72.9%) and 88.1% (95% CI 77.8–94.7%) respectively.

In 38 cases (29.7%) we observed the combination of negative LUS and negative PCT (<0.25 ng/ml). A false negative combination of LUS/PCT test was observed in 2 cases (1.6%). In one of these two patients the final diagnosis was infective endocarditis and heart failure associated to pneumonia. The other patient had a final diagnosis of pneumonia complicated by heart failure and non-ST elevated myocardial infarction. The derived sensitivity of negative LUS/PCT test was 96.7% (95% CI 88.6–99.5%) with a significant superiority to LUS alone and PCT alone (*p* < 0.05 for both). Negative predictive value (NPV) and negative likelihood ratio (LR) were 94.7% (95% CI 88.2–99.2%) and 0.06 (95% CI 0.02–0.24) respectively. In 24 patients (18.8%) we observed the combination of positive LUS and positive PCT (>0.5 ng/ml). Out of these, four were false positive (3.1%). The derived specificity, positive predictive value (PPV) and positive LR were 94% (95% CI 85.4–98.3%), 83.3% (95% CI 62.6–95.2%) and 5.49 (95% CI 1.99–15.17) respectively. Specificity of the LUS/PCT test was not significantly different when compared to LUS alone (*p* = 0.125).

In 46 patients (35.9%), CXR was negative for pneumonia and PCT was <0.25 ng/ml. False negative cases where 12 (9.4%). The derived sensitivity, NPV and negative LR were 80.3% (95% CI 68.1–89.4%), 73.9% (95% CI 58.9–85.7%) and 0.39 (95% CI 0.22–0.68) respectively. In 16 patients (12.5%) CXR was positive for pneumonia and PCT was >0.5 ng/ml (16, 12.5%), with one false positive case (0.8%). The derived specificity, PPV and positive LR were 98.5% (95% CI 91.9–99.7%), 93.7% (95% CI 69.7–98.9%) and 16.48 (95% CI 2.24–121.05%) respectively.

The sensitivity of negative LUS/PCT (<0.25 ng/ml) was significantly higher than negative CXR/PCT (96.7 vs 80.3%,* p* < 0.05), whereas the specificity of positive LUS/PCT (>0.5 ng/ml) was not significantly different to positive CXR/PCT (94 vs 98.5%,* p* = 0.68). Overall test characteristics of LUS, CXR and PCT and of LUS and CXR combined with PCT are reported in Tables 3 and 4.

**Table 3:** Diagnostic performance of lung ultrasonography, chest radiograph and procalcitonin for the diagnosis of pneumonia

	Sens % (95% CI)	Spec % (95% CI)	PPV % (95% CI)	NPV % (95% CI)	+LR % (95% CI)	−LR (95% CI)
LUS	85.2 (73.8–93)	88 (77.8–94.7)	86.7 (75.4–94)	86.8 (76.3–93.7)	7.14 (3.70–13.79)	0.17 (0.09–0.31)
CXR	60.7 (47.3–72.9)	88.1 (77.8–94.7)	82.2 (67.9–92)	71.1 (60.1–80.5)	5.08 (2.57–10.04)	0.45 (0.32–0.62)
PCT ≥ 0.25	73.8 (61–84.2)	47.8 (35.4–60.3)	56.2 (44.7–67.3)	66.7 (51.6–79.6)	1.41 (1.07–1.86)	0.55 (0.34–0.90)

*Sens* sensitivity; *Spec* specificity; *PPV* positive predictive value; *NPV* negative predictive value; *+LR* positive likelihood ratio; *−LR* negative likelihood Ratio; *95% CI 95%* confidence interval; *CXR* chest X-ray; *LUS* lung ultrasonography; *PCT* procalcitonin value express in ng/ml

**Table 4:** Diagnostic performance of lung ultrasonography and chest radiograph combined with procalcitonin for the diagnosis of pneumonia

	Sens % (95% CI)	Spec % (95% CI)	PPV % (95% CI)	NPV % (95% CI)	+LR (95% CI)	−LR (95% CI)
Positive LUS or PCT ≥0.25	96.7 (88.6–99.5)	53.7 (41.1–66)	65.6 (54.8–75.3)	94.7 (82.2–99.2)	2.09 (1.61–2.72)	0.06 (0.02–0.24)
Positive LUS and PCT >0.5	32.8 (21.3–46)	94 (85.4–98.3)	83.3 (62.6–95.1)	60.6 (50.5–70)	5.49 (1.99–15.17)	0.71 (0.59–0.86)
Positive CXR or PCT ≥0.25	80.3 (68.1–89.4)	50.7 (38.2–63.2)	59.8 (48.3–70.4)	73.9 (58.9–85.7)	1.63 (1.2–2.1)	0.39 (0.22–0.68)
Positive CXR and PCT >0.5	24.6 (14.5–37.3)	98.5 (91.9–99.7)	93.7 (69.7–98.9)	58.9 (49.2–68.1)	16.48 (2.24–121.05)	0.77 (0.66–0.89)

*Sens* sensitivity; *Spec* specificity; *PPV* positive predictive value; *NPV* negative predictive value; *+LR* positive likelihood ratio; *−LR* negative likelihood Ratio; *95% CI* 95% confidence interval; *CXR* chest X-ray; *LUS* lung ultrasonography; *PCT* procalcitonin value express in ng/ml; or means that at least one test was positive; and means that both tests were positive

**Fig. 2:**
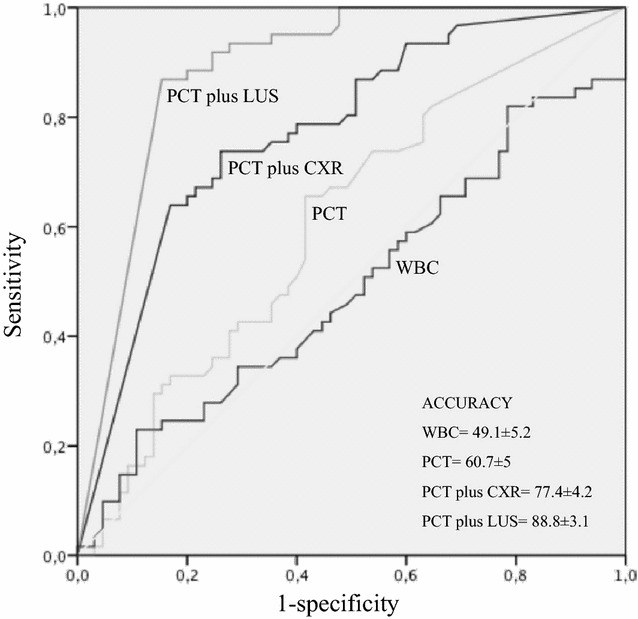
ROC curve of WBC, PCT, LUS and CXR and their combination. *WBC* white blood cells; *PCT* procalcitonin; *LUS* lung ultrasonography; *CXR* chest X-ray

According to ROC curve, the diagnostic accuracy of LUS associated with PCT (88.8% ± 3.1) was higher than the total count of white blood cells (WBC) (49.1% ± 5.2), PCT alone (60.7% ± 5) and CXR associated with PCT (77.4% ± 4.2) (Fig. 2).

## Discussion

To our knowledge, this is the first study evaluating the diagnostic performance of LUS combined with PCT for the diagnosis of pneumonia in the ED. Research on the diagnosis of pneumonia is usually hampered by difficulties in obtaining a systematic comparison with CT scan. The lack of a gold standard that supports the visual diagnosis of pneumonia, may lower the significance of many studies. One strength of our study is that the final diagnosis of pneumonia in all patients is supported by a post hoc review of the clinical chart, and based on the detection of typical consolidations at chest CT read by an expert radiologist. In many previous studies LUS showed to be an accurate bedside tool for the diagnosis of pneumonia [8, 15–17]. In our study LUS alone ruled in pneumonia with a good positive likelihood ratio and ruled out this condition with a moderate negative likelihood ratio. The superiority of LUS over CXR is reported in literature [9]. In our study, despite the two methods showed similar specificities, the sensitivity of LUS was superior to CXR. We have to consider that in 8% of patients, pulmonary ultrasound examination was limited only to the anterior-lateral areas and that 33% of all false negative cases belong to this group. In these patients, most dependent pulmonary areas were not scanned and consolidations were not detected and this was the cause of a reduction in overall sensitivity of LUS for the diagnosis of pneumonia.

Procalcitonin is an emerging biomarker, whose practical application is increasingly common, not only in the diagnosis and follow-up of sepsis but also in pneumonia [18]. A PCT value below the threshold level makes unlikely a severe pneumonia with bacteraemia and is used to withhold antibiotic therapy [19–21]. Müller et al. analysed data from two randomized prospective studies with a total of 545 patients with suspected infection of the lower respiratory tract. In their post-hoc analysis, they found that the diagnostic accuracy to predict radiographically suspected community acquired pneumonia was significantly higher for PCT (87%) than for WBC (64%) [22]. In our study, the diagnostic accuracy of PCT, although inferior to the performance obtained by Müller et al., was still higher than WBC (49%).

The lower accuracy of PCT found in our study compared with the study by Müller may be due to a different selection of patients and to the different gold standard used for the diagnosis of pneumonia.

Despite its known role in the confirmation of bacteraemia in sepsis, in our study PCT alone was not sufficiently accurate in the diagnosis of pneumonia. This can be explained considering that while the final diagnosis of pneumonia was only confirmed in presence of typical clinical signs with at least one typical consolidation detected at chest CT, we cannot confirm that in our patients the radiologic consolidations were always due to a bacterial infection with bacteraemia. The majority of patients with a final diagnosis of pneumonia were in a stable condition and showed an isolated pulmonary infection not necessarily associated with bacteraemia. It is well known that in cases of localized organ infections, PCT is usually lower as compared to septicaemia with positive blood cultures [23].

No previous studies evaluated the association of LUS and PCT for the bedside diagnosis of pneumonia in the ED. However, a recent study by Zagli et al. performed on critically ill patients in the intensive care unit, evaluated a new score, CEPPIS (chest echography and procalcitonin pulmonary infection score), based on a combination of PCT and LUS for the diagnosis of ventilator associated pneumonia (VAP) [24]. The new proposed score differed from the traditional clinical pulmonary infection score (CPSI) basically in two items: WBC was replaced by PCT and CXR by LUS. The study showed that sensitivity of CEPPIS (80.5%) was superior then CPSI (39.8%) for the diagnosis of VAP, while specificity was similar (85.2 vs 83.3%). There are many methodological differences between the study of Zagli et al. and our study, but both showed that the sensitivity of LUS/PCT is superior to other diagnostic approach based on CXR in the diagnosis of pneumonia.

In our study the association of a negative LUS examination with a PCT level <0.25 ng/ml significantly improved sensitivity of LUS for the diagnosis of pneumonia, reducing the occurrence of false negative diagnoses and decreasing the negative LR. We can argue that the clinician, in approximately one-third of “difficult” diagnosis in patients with unexplained respiratory symptoms, may rule-out pneumonia with a sufficient level of certainty by combining LUS and PCT. On the other hand, our study showed that when LUS visualizes a consolidation with the typical ultrasonography features, the specificity and positive predictive value for the diagnosis of pneumonia is not significantly influenced by the addiction of PCT.

Lung ultrasonography is a powerful, cheap, easy to perform, and safe tool for the diagnosis of many pulmonary conditions at bedside and its use is exponentially increasing among specialists and general practitioners in and out of hospitals. Moreover, the diagnostic accuracy of LUS for pneumonia is at least as good as CXR in different settings and patients. Considering that a point-of care PCT measurement will become available for clinical practice [10], the results of this preliminary study opens new perspectives for improving the diagnostic approach to pneumonia at the bedside, by combining LUS and PCT in the general population. Further studies could also evaluate the usefulness of this combined diagnostic strategy in some subgroup of patients where CXR is less accurate or contraindicated such as bedridden patients in particular patients in invasive and not invasive ventilation, immunocompromised patients and pregnant women. Furthermore, using multiple biomarkers combined with LUS could further increase diagnostic accuracy of physicians and be useful to triage patients at the bedside in many different clinical scenarios[25].

### Study limitations

A questionable characteristic of our protocol is that patients were not enrolled on the basis of a direct suspicion for pneumonia. We concentrated our study on patients with respiratory complains, who demanded CT scan for ambiguous diagnosis. In case of suspected uncomplicated lung infection, where the diagnosis is mainly based on clinical findings and CXR, it is unusual to undergo CT scans. Thus, our specific patient population does not allow generalization of our results. However, a systematic application of CT studies in all suspected low respiratory tract infection is not feasible because it does not represent a standard of care, and it is not ethical for its radiation burden and not sustainable for high cost. Another limitation of our study, is that the final diagnosis of pneumonia was based on the detection of a consolidation at chest CT, while the possibility of interstitial pneumonia was not considered. We have also to consider that it is not known if the consolidation identified at chest CT was due to bacterial, viral or other germ infection. Regarding the comparison between LUS and CXR, a consideration should be done on the criteria of enrolment. In fact, the patients enrolled in the study underwent chest CT once the physician was aware of CXR results, and this could have selected a population with not diagnostic CXR. Thus, this criteria probably affected the low sensitivity of CXR. However, in the real practice, the role of LUS is often to confirm negative chest films in patients with high suspicion of pneumonia. Thus, the difference in sensitivity between the two methodologies is still confirmed. Indeed, the fact that in our study the majority of CXR were not performed in the up-right position, may have influenced more significantly the comparison between the two methods. The anterior-posterior radiographic view is undoubtedly less accurate for the study of the lungs. However, the evaluation of sub-optimal chest films is largely representative of the real daily practice and the main cause of inconclusive CXR studies. Finally, the lack of systematic and consecutive PCT assays in the population studied may have biased our results. Indeed, a PCT assay was only based on the personal judgement of the attending physician facing challenging diagnostic situations and was independent from the study protocol. However, these situations are exactly those deserving more accurate and sometimes alternative diagnostic tools, that is the possible future role of a combined LUS/PCT protocol.

## Conclusions

The combination of LUS and PCT showed a high sensitivity for the diagnosis of pneumonia in patients presenting with respiratory complaints of uncertain origin that underwent chest CT and the sensitivity of this combination was significantly higher when compared with the sensitivity of LUS alone and PCT alone.
